# RNF128 Promotes Invasion and Metastasis Via the EGFR/MAPK/MMP-2 Pathway in Esophageal Squamous Cell Carcinoma

**DOI:** 10.3390/cancers11060840

**Published:** 2019-06-18

**Authors:** Jing Gao, Yang Wang, Jie Yang, Weixia Zhang, Kun Meng, Yue Sun, Yangjia Li, Qing-Yu He

**Affiliations:** Key Laboratory of Functional Protein Research of Guangdong Higher Education Institutes, Institute of Life and Health Engineering, College of Life Science and Technology, Jinan University, Guangzhou 510632, China; 13304502656@163.com (J.G.); wangyang8857@gmail.com (Y.W.); sciencetrapper@foxmail.com (J.Y.); tobedonezwx@163.com (W.Z.); mengk_123@126.com (K.M.); SunY1833147899@163.com (Y.S.); murray_95@126.com (Y.L.)

**Keywords:** ESCC, RNF128, migration, invasion, MMP-2

## Abstract

*Background:* The prognosis of esophageal squamous cell carcinoma (ESCC) is generally poor, and the identification of molecular markers related to the regulation of ESCC invasion and migration is important. *Methods and Results:* In this study, we report that ring finger protein-128 (RNF128) enhances the invasiveness and motility of ESCC cells by using transwell assays and Western blotting. A xenograft nude mouse model showed that RNF128 promotes the metastasis of ESCC cells in the lung. A signal pathway analysis identified the epidermal growth factor receptor (EGFR)/mitogen-activated protein kinase (MAPK)/matrix matalloproteinases 2 (MMP-2) cascade as a mediator of RNF128-induced enhancement of ESCC progression. Inhibition experiments using inhibitors of EGFR, ERK kinase (MEK)/extracellular-signal-regulated-kinase (ERK), and MMP-2 reversed this progression. Co-immunoprecipitation demonstrated that RNF128 promotes the activation of the EGFR/ERK/MMP-2 pathway by interacting with p53 and p53 interacting with EGFR. *Conclusion:* Our results establish the functional role of RNF128 in driving the invasion and metastasis of ESCC through the EGFR/MAPK/MMP-2 pathway, implicating its potential as a candidate therapeutic target and prognostic biomarker for ESCC.

## 1. Introduction

Esophageal cancer has the ninth-highest cancer incidence and is the sixth leading cause of cancer death worldwide, and is therefore a major global health challenge [1]. Globally, the most common histological type of esophageal cancer is esophageal squamous cell carcinoma (ESCC) [1,2,3]. ESCC has a particularly poor prognosis and is often diagnosed in the later stages of the disease, and more than half of patients have distant metastases [4]. This has led to a decline in its 5-year survival rate. In recent years, despite advances in diagnosis and treatment, the 5-year survival rate is still between 15% and 20% [5]. Although many genes have been known to be involved in the invasion and metastasis of ESCC, there is still a lack of effective strategies to control the progression of the disease. Therefore, it is necessary to further identify key genes as tumor markers and molecular targets to help resolve the malignant processes of ESCC.

Ring finger protein-128 (RNF128, also known as Grail) is a type I transmembrane protein that localizes to the transferrin-recycled endocytic pathway and is homologous to the RING finger protein [6,7]. It was initially determined that RNF128 mRNA is induced in non-reactive T helper 1 (Th1) cells [6]. Activation of NFATc1 (a nuclear factor of activated T cell 1) homodimer via calcium signaling is followed by activation of RNF128 mRNA expression [8,9]. RNF128 controls Th2 development to play an important role through a negative feedback loop [10,11]. A previous report found that RNF128 is a p53-interacting glycoprotein that functions in p53-induced apoptosis under stress conditions [12]. The important role of p53 in cancer is well known, but the role of RNF128 in tumors remains unknown. Previously, two studies have shown that RNF128 undertakes critical functions in addition to energy regulation. The first study clarified the important function of RNF128 in nutrient metabolism, and the other study identified the key role of RNF128 in non-lymphocyte development [13,14]. To our knowledge, this work is the first report to clarify the relationship between RNF128 and ESCC invasion and metastasis.

In this study, we aimed to investigate the role of RNF128 in ESCC and explore its functional mechanism. Our results show that RNF128 facilitates ESCC invasion in vitro and metastasis in vivo by increasing EGFR and MMP-2 expression. Further investigation indicated that RNF128 regulates MMP-2 by activating the EGFR/MAPK/MMP-2 signaling pathway. Thus, RNF128 may serve as a new therapeutic target or clinical biomarker for metastatic ESCC.

## 2. Results

### 2.1. RNF128 Promoted ESCC Cell Migration and Invasion In Vitro

We first examined the expression of RNF128 in nine ESCC cells using Western blotting (Figure S1A). The result showed that the expression of RNF128 had no significant difference among the nine cell lines including EC109 and KYSE270 with a low metastatic grade, and KYSE30, KYSE520, and EC9706 with a higher metastatic grade. To ascertain whether there is a causative relationship between RNF128 expression and an altered cancer phenotype in ESCC, we selected KYSE150 and KYSE410 cells with relatively low RNF128 expression to construct cell lines with RNF128 stably overexpressing. Then, we conducted a study on the growth and proliferation of ESCC cells. As shown in Figure S1B,C, RNF128 overexpression did not make KYSE150 and KYSE410 cells have a stronger ability for clone formation and cell proliferation. 

**Figure 1:**
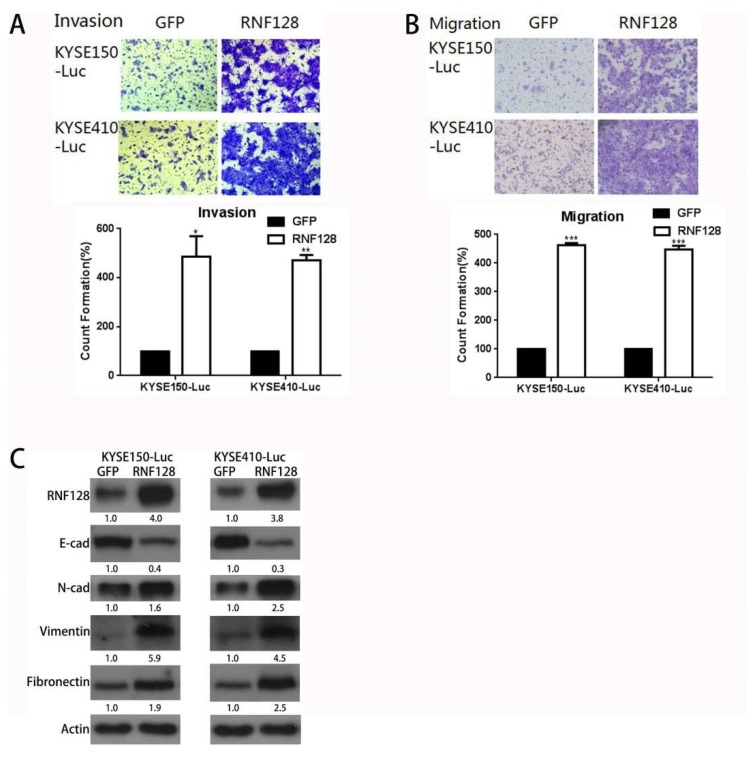
Ring finger protein-128 (RNF128) overexpression promoted esophageal squamous cell carcinoma (ESCC) cell migration and invasion in vitro. RNF128-overexpression and control cells invaded through a matrigel-coated membrane (**A**) or migrated through a membrane (**B**). (**C**) RNF128, E-cadherin, N-cadherin, vimentin, and fibronectin expression in RNF128-overexpression and control cells were confirmed using Western blotting. Magnification for (**A**): ×40, magnification for (**B**): ×40.

Next, we conducted a study on the invasion and migration of the ESCC cells. Cell lines stably overexpressing RNF128 clearly acquired enhanced cell migration and the ability to invade through the extracellular matrix coating (Figure 1A,B). The decreased levels of E-cadherin, in combination with the upregulation of N-cadherin, vimentin, and fibronectin after RNF128 overexpression, suggested that RNF128 regulates the progress of epithelial–mesenchymal transition (EMT) (Figure 1C). 

**Figure 2:**
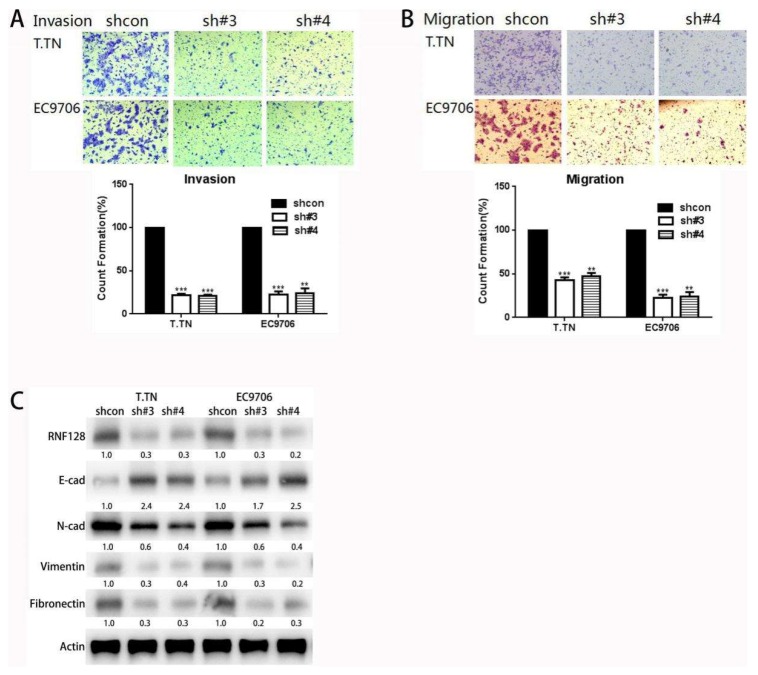
RNF128 knockdown repressed ESCC cell migration and invasion in vitro. Cell invasion (**A**) and migration (**B**) assays and quantification were performed in RNF128 knockdown and control cells, respectively. (**C**) E-cadherin, N-cadherin, vimentin, and fibronectin expression in RNF128-knockdown and control cells were confirmed using Western blotting. Magnification for (**A**): ×40, magnification for (**B**): ×40.

We then chose T.TN and EC9706 cells for a knockdown study because RNF128 is relatively highly expressed in these cells as compared to KYSE410 and KYSE150 cells. The successful knockdown of RNF128 was validated at the protein level (Figure S1D). In the cell invasion assay, we found that when RNF128 expression was knocked down, the invasive ability of cells was reduced compared to control cells (Figure 2A). In the cell migration assay, we observed a decrease in cell migration ability as RNF128 expression decreased (Figure 2B). Then, we observed the suppression of the EMT progress via RNF128 knockdown with the decreased levels of N-cadherin, vimentin, and fibronectin (Figure 2C). Collectively, these data indicated that RNF128 contributes to the augmented invasion and metastasis of ESCC cells.

### 2.2. Transcriptome Sequencing Identified the Activation of EGFR/MAPK/MMP-2 Signaling Induced by RNF128

**Figure 3:**
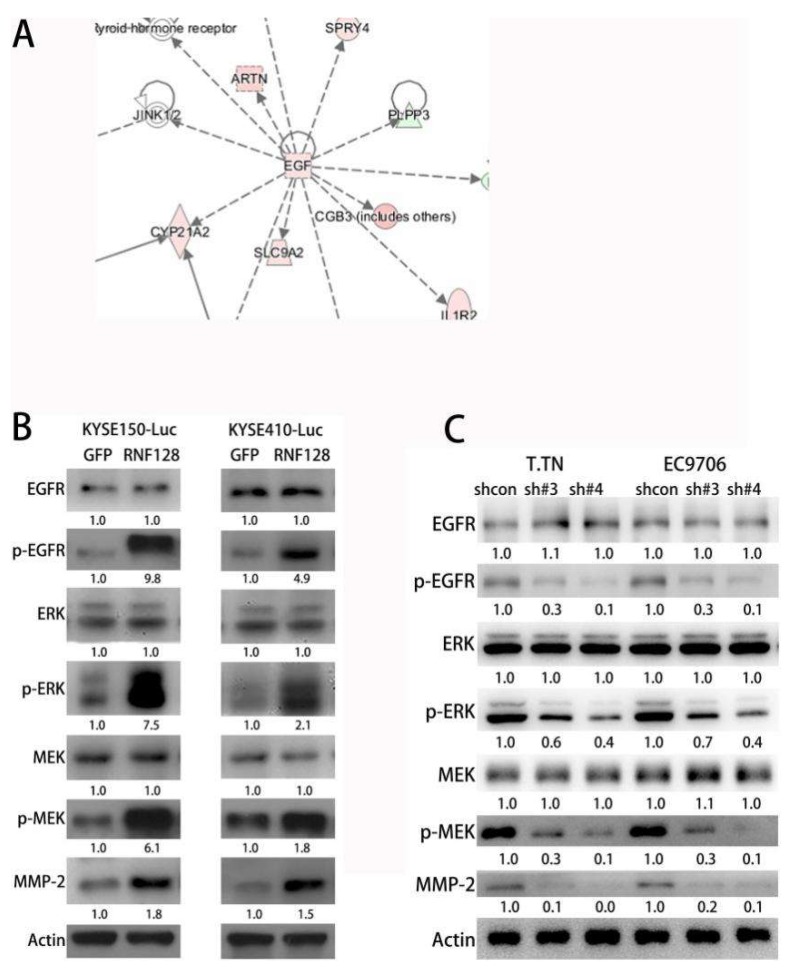
RNF128 enhanced MMP-2 expression through activating the EGFR/MAPK/MMP-2 pathway. (**A**) IPA analysis of genes differentially expressed between RNF128-overexpressing KYSE150 and KYSE150-Luc cells, suggesting the EGF pathway was involved. (**B**) Western blot analysis for the expression of p-EGFR, total EGFR, p-ERK, total ERK, p-MEK, total MEK, and MMP-2 in RNF128-overexpressing cells. (**C**) Western blot analysis for the expression of p-EGFR, total EGFR, p-ERK, total ERK, p-MEK, total MEK, and MMP-2 in RNF128 knockdown cells.

To better understand the molecular events, we used transcriptome sequencing (ANORAD, Jiangsu, China) to screen for differentially regulated genes in KYSE150-Luc and KYSE150-RNF128 cells (Figure 3A). On this basis, we further investigated the molecular mechanism involved in the effect of RNF128 in ESCC cells. Ingenuity pathway analysis (IPA) (Ingenuity Systems, Redwood City, CA, USA) was used to characterize the canonical pathways in which the differentially expressed genes participated, showing the up-regulation of EGF and MMP-2 and the p53 interaction with RNF128 and EGFR (Figure S2A–D). 

Previous studies have shown that EGFR activation causes downstream Ras-Raf-MEK-ERK signaling activation leading to changes in MMP expression [15,16,17]. Given the potential role of RNF128 in EGFR signaling, we hypothesized that overexpression of RNF128 would activate EGFR phosphorylation, thereby activating ERK1/2, and subsequently upregulating MMP-2 expression. In this regard, p-EGFP, p-MEK, p-ERK, and MMP-2 were found to be upregulated in ESCC cells with stably overexpressed RNF128 (Figure 3B). Conversely, these phosphoproteins were downregulated in ESCC cells with stably repressed RNF128 (Figure 3C), suggesting that RNF128 mediated the EGFR/MAPK/MMP-2 pathway in ESCC cells. These results demonstrated that RNF128 could upregulate EGFR phosphorylation, which subsequently drove downstream MAPK/MMP-2 signaling.

### 2.3. RNF128 Drove ESCC Cell Invasion and Metastasis through the EGFR/MAPK/MMP-2 Signaling Pathway

**Figure 4:**
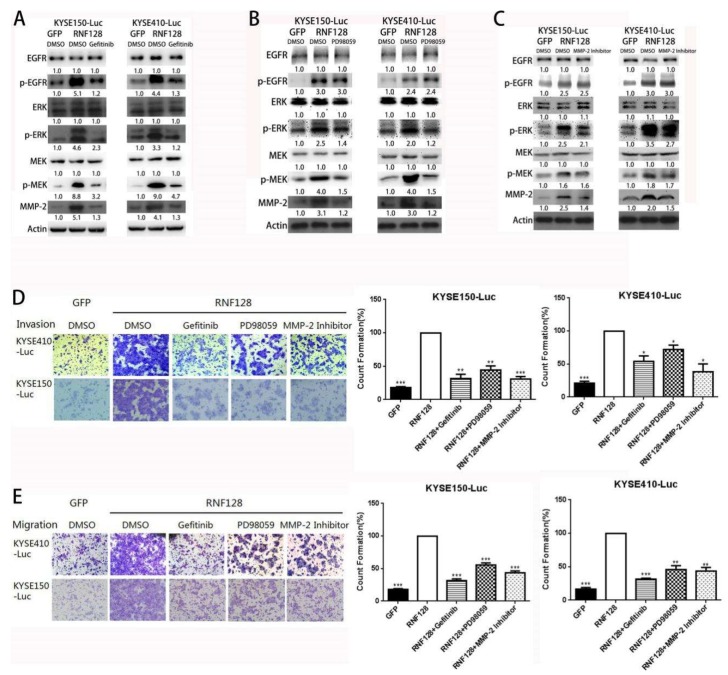
RNF128 promoted the activation of the EGFR/ERK/MMP-2 pathway. RNF128-overexpression cells were pretreated with gefitinib (1 µM), PD98059 (10 µM), or MMP-2 inhibitor (1 µM) for 24 h. (**A**–**C**) Western blot analysis of the expression levels of EGFR, p-EGFR, ERK, p-ERK, MEK, p-MEK, and MMP-2. Cell invasion (**D**) and migration (**E**) were evaluated after treatment for 24 h. Magnification for (**D**): ×40, magnification for (**E**): ×40.

To further validate the role of the epidermal growth factor receptor (EGFR)/mitogen-activated protein kinase (MAPK)/matrix matalloproteinases 2 (MMP-2) activation in RNF128-induced metastasis, we analyzed the impact of introducing gefitinib (an inhibitor of EGFR), PD98059 (an inhibitor of MEK/ERK), and MMP-2 inhibitor into ESCC cells with stably overexpressed RNF128. Successful suppression of the EGFR/MAPK/MMP-2 pathway following the addition of gefitinib in ESCC cells with stably overexpressed RNF128 was confirmed by the diminished expression of p-EGFR, p-MEK, p-ERK, and MMP-2 proteins (Figure 4A). Inhibition with PD98059 attenuated the expression of p-MEK, p-ERK, and MMP-2 (Figure 4B). As shown in Figure 4C, the MMP-2 inhibitor significantly inhibited the expression of MMP-2 in RNF128-overexpression cells. Subsequently, we also found that the migration and invasion abilities of KYSE150-RNF128 and KYSE410-RNF128 cells were suppressed by the treatment with gefitinib, PD98058, and MMP-2 inhibitor (Figure 4D,E). Collectively, these data indicated that RNF128 acted upstream of the EGFR/MAPK/MMP-2 pathway to promote ESCC cell invasion and metastasis.

### 2.4. RNF128 Promoted the Activation of EGFR/MAPK/MMP-2 Pathway by Interacting with p53 and p53 Interacting with EGFR

**Figure 5:**
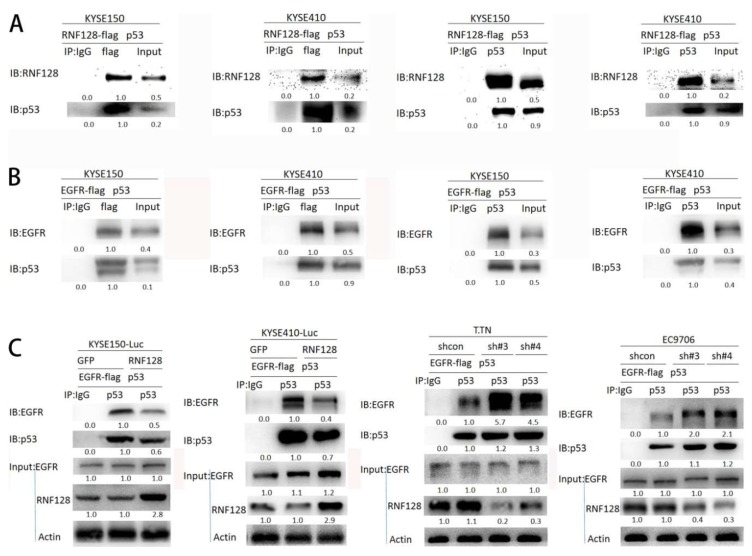
RNF128 promoted the activation of the EGFR/MAPK/MMP-2 pathway by interacting with p53 and p53 interacting with EGFR. (**A**) p53’s binding with RNF128 was validated using a CoIP assay. (**B**) A flag-tagged EGFR plasmid and a p53 plasmid were transfected into KYSE150 and KYSE410 cells. CoIP demonstrated that EGFR and p53 could be coprecipitated. (**C**) A flag-tagged EGFR plasmid and a p53 plasmid were transfected into RNF128 overexpressed or knocked-down ESCC cells. CoIP demonstrated that EGFR and p53 could be coprecipitated.

p53 has been indicated to interact with RNF128 and EGFR via functional protein association networks (STRING) analysis (Figure S2D). RNF128 has previously been reported to directly interact with p53 [12]. Two other studies have also found that p53 interacts with EGFR [18,19]. Our Co-immunoprecipitation (CoIP) assays confirmed that p53 exists in complexes precipitated with antibody against a flag (RNF128) as compared with control IgG (Figure 5A). p53’s binding with RNF128 was further validated using CoIP assay with an antibody against p53 (Figure 5A). To further confirm whether p53 interacts with EGFR, a flag-tagged EGFR plasmid and a p53 plasmid were transfected into KYSE150 and KYSE410 cells. The CoIP experiment demonstrated that EGFR and p53 could be coprecipitated, indicating the interaction between them (Figure 5B). These results imply that RNF128 promoted the phosphorylation of EGFR by interacting with p53. 

To further support this conclusion, we transiently transfected the p53 plasmid and the flag-tagged EGFR plasmid into the cells with or without RNF128 overexpression and then performed the CoIP assay. As shown in Figure 5C, the interaction between EGFR and p53 was reduced with RNF128 overexpression, but increased in RNF128 knockdown cells. Therefore, we concluded that RNF128 inhibited the interaction of p53 and EGFR by interacting with p53, activating the EGFR pathway. 

### 2.5. RNF128 Promoted Tumor Metastasis In Vivo

**Figure 6:**
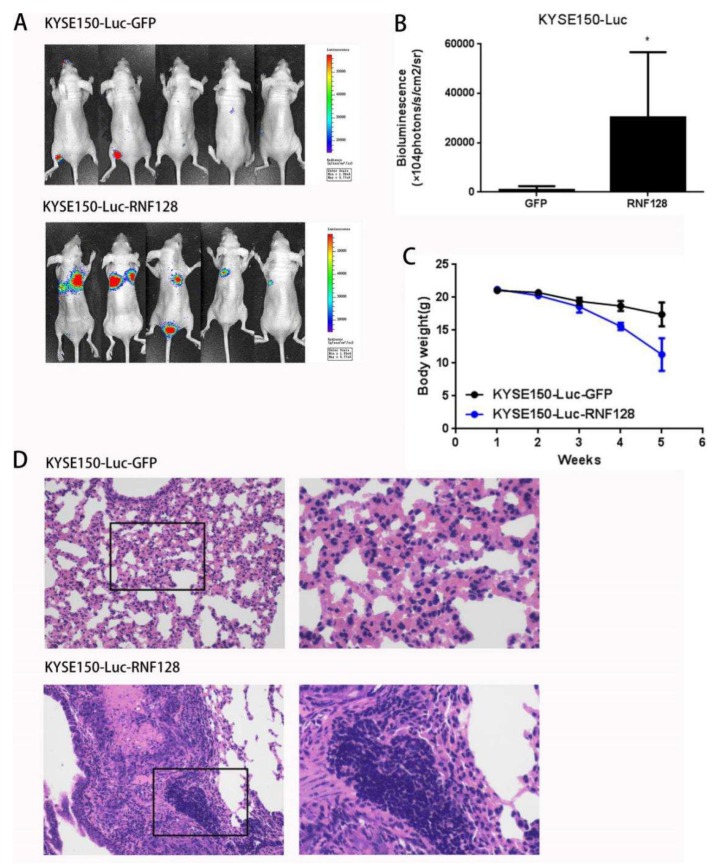
RNF128 promoted tumor metastasis in vivo. (**A**,**B**) Bioluminescent imaging and quantification of RNF128-overexpressing and control cells in mice. (**C**) Body weight of nude mice with the RNF128-overexpressing and control cells during the experimental period. (**D**) Hematoxylin and eosin (H&E) staining of the lungs collected from the mice of the RNF128-overexpressing and control groups. Magnification for (D): left, ×200; right, ×500.

To further substantiate the role of RNF128 in driving ESCC metastasis, in vivo experiments with tail vein metastasis models were also conducted. ESCC cells with stably overexpressed RNF128 displayed a superior ability to metastasize to the lung following cell injection through the tail vein as compared with controls, as supported by stronger bioluminescence signals in the lung (Figure 6A,B). Furthermore, a significant difference between the RNF128-overexpressed animals and control groups was observed in terms of body weight (Figure 6C). Histologic analysis revealed significantly more and larger metastatic foci in the harvested lung tissues of mice injected with ESCC cells with overexpressed RNF128 (Figure 6D). These observations suggest a role for RNF128 in the regulation of metastasis in ESCC.

## 3. Discussion

**Figure 7:**
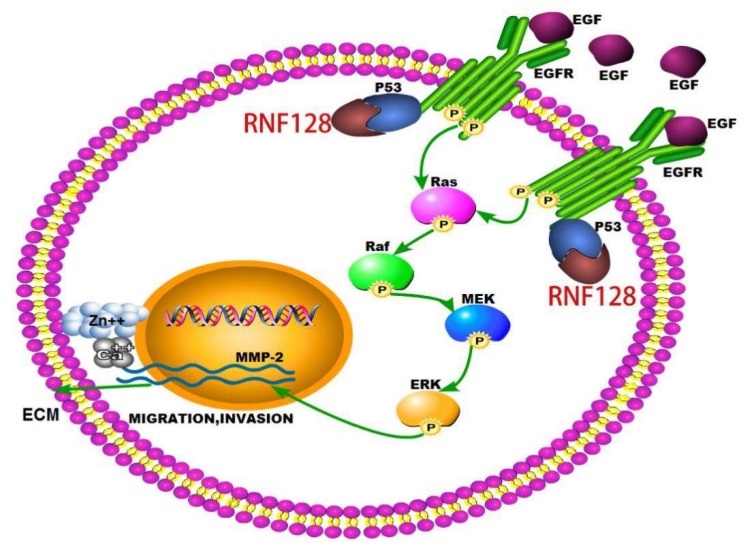
Schematic diagram summarizing the RNF128-induced EGFR/MAPK/MMP-2 pathway. RNF128 is highlighted in red.

Rapid invasion and migration of ESCC cells are important factors leading to poor prognosis in ESCC patients. Therefore, it is important to find molecules that inhibit the invasion and metastasis of ESCC cells to improve the prognosis of ESCC. In the present study, we examined the role of RNF128 in ESCC growth and metastasis. We found that overexpression of RNF128 promoted ESCC cell migration and invasion, and that RNF128 silencing decreased ESCC invasion and metastasis in vitro. However, overexpression of RNF128 did not significantly increase cell viability and proliferation of ESCC cells. Further analyses showed that RNF128 positively regulated MMP-2 expression by activating the EGFR/MAPK/MMP-2 signaling pathway in ESCC. CoIP demonstrated that RNF128 interacted with p53, and p53 interacted with EGFR and then promoted the activation of the EGFR/MAPK/MMP-2 pathway (Figure 7). Intravenous injection of ESCC cells into nude mice confirmed that RNF128 enhanced the possibility of the lung metastasis of ESCC cells.

Members of the EGF family first bind to their homologous cell surface receptors, which form a homodimer or heterodimer upon binding to an EGF-like ligand and then activate the receptor via autophosphorylation [15]. Upon receptor activation, different downstream adaptor molecules are recruited, triggering activation of multiple downstream signal transduction pathways, such as MAPK, PI3K-AKT, JAK-STAT, and phospholipase C cascades [15]. Different signaling pathways are activated by different EGF ligands, which drive various transcription factors into the nucleus and regulate various cellular events such as proliferation, apoptosis, invasion, and migration [15]. In this connection, when the activity of EGFR was inhibited in our ESCC cells, the invasive phenotype of RNF128-induced cell movement was reversed. The same results were obtained with the use of MAPK pathway inhibitors. This observation provides a mechanistic rationale for the relationship between RNF128 and ESCC progression via EGFR-MARK signaling.

MMPs are a family of structurally related zinc and calcium-dependent endopeptidases that degrade extracellular matrices, thereby enhancing the progression of epithelial–mesenchymal transition [20]. MMP-2 is a key enzyme involved in the proteolysis of the major components of the basement membrane and is known to have a clear correlation with tumor invasion and metastasis [21,22]. Previous reports also revealed the link between MMP-2 expression and the activation of EGFR and Raf-MEK-ERK signaling [15,16,17]. Our IPA analysis suggested that RNF128 may modulate EGF and MMP-2, activating EGFR signaling. With this in mind, we here verified that the increased expression of RNF128 in ESCC cells promotes MMP-2 expression levels, indicating that RNF128 has the potential to induce ESCC invasion and metastasis. We further found that RNF128 promotes phosphorylation of EGFR, and that inhibition of EGFR phosphorylation suppresses RNF128-induced MMP-2 expression, confirming that the EGFR/MAPK/MMP-2 pathway is involved. 

In addition, whether RNF128 also regulates other signaling pathways is worth exploring further. Two studies from Sheikh et al. and Ludes-Meyers et al. [18,23] demonstrated that the true target of p53 is the promoter of EGFR, and that p53 regulates the EGFR promoter from multiple independent sites. Our further goal is to study the crosstalk between RNF128, p53, and EGFR-mediated signal transduction pathways, and the molecular basis by which RNF128 regulates phosphorylation of EGFR. 

Our current findings demonstrate the role of RNF128 in promoting ESCC invasion and metastasis, both in vivo and in vitro. RNF128 regulates the EGFR/MAPK/MMP-2 pathway to drive these aggressive cancer features in ESCC. Given the potent function of RNF128 in the malignant progression of ESCC, RNF128 could be further explored to be a novel therapeutic target for metastatic ESCC. 

## 4. Materials and Methods

### 4.1. Cell Lines and Culture

All cells including KYSE150, KYSE510, KYSE410, KYSE520, KYSE270, KYSE30, EC9706, EC109 and T.TN were grown in 1640 (RPMI-1640) medium containing 10% fetal bovine serum (Gibco, Grand Island, NY, USA) and cultured in an incubator containing 5% carbon dioxide at 37 °C.

### 4.2. Transduction

The RNF128 shRNA expression vector and the shRNA non-target control were purchased from TransheepBio (Shanghai, China). RNF128 lentiviral overexpression or empty vector control plasmids were gifts from Dr. Bin Li (College of Life Science and Technology, Jinan University, Guangzhou, China). Sequences were transfected into 293T cells, packaged using Lentiviral Packaging Mix (Life technologies corporation Gaithersburg, MD, USA) and used to infect ESCC cells to establish cells constitutively repressing or expressing RNF128. Stable clones were selected with puromycin (1 μg/mL).

The plasmids expressing p53 and RNF128-flag were gifts from Dr. Bin Li (College of Life Science and Technology, Jinan University). The plasmids expressing an EGFR-flag were purchased from TransheepBio. The cells were grown in RPMI-1640 medium, grown to 50%, and then transfected using Lipofectamine 3000 (Life technologies corporation Gaithersburg, MD, USA) according to the manufacturer’s instructions.

### 4.3. Western Blot Analysis 

The cells were lysed for 30 min and then centrifuged at 13,000× *g* for 30 min at 4 °C. The quantified protein was mixed in proportion with the protein loading buffer and boiled at 95 °C for 10 min. The sample was electrophoresed and subsequently transferred to a polyvinylidene difluoride (PVDF) membrane. The non-fat milk powder was diluted with Tween-20 Tris buffered saline (TBST) for 1 h at room temperature, then incubated with primary antibody overnight, washed with TBST, and then incubated with the corresponding secondary antibody for one hour at room temperature. The reaction was visualized using electrochemiluminescence (ECL, Bio-Rad, Hercules, CA, USA) and detected via exposure to an autoradiographic film. Densitometry readings/intensity ratio of each band were included in all Western blot figures. Whole blots showing all the bands with molecular weights were provided as Figures S3 and S4.

The primary antibodies used included E-cadherin and N-cadherin from BD Pharmingen (San Diego, CA, USA); vimentin, EGFR, P-EGFR, ERK, P-ERK, MEK, and P-MEK from Cell Signaling Technology (Danvers, MA, USA); fibronectin, p53, and MMP-2 from Proteintech (Rosemont, IL, USA); actin from Transgen Biotech (Beijing, China); PD98059 from Selleck (Houston, TX, USA); MMP-2 inhibitor from Cayman (Ann Arbor, MI, USA); flag from Sigma (St. Louis, MO, USA); and gefitinib was kindly provided by Dr. Bin Li (College of Life Science and Technology, Jinan University).

### 4.4. In Vitro Transwell Assay of Cell Migration and Invasion

Migration: 2 × 10^5^ cells were resuspended in serum-free medium and added to the top chamber, and then a medium containing 10% FBS was added to the bottom chamber. After incubation for 24 h at 37 °C, cells that migrated were fixed with methanol and stained with 0.2% crystal violet, and then quantified by four random fields under a microscope.

Invasion: 100 μL of Matrigel (Corning Incorporated, Corning, NY, USA) was added to each chamber and placed at 37 °C for 20 min. A total of 2 × 10^5^ cells were resuspended in serum-free medium and added to the top chamber, and then medium containing 10% FBS was added to the bottom chamber. After incubation for 24 h at 37 °C, cells that invaded through the Matrigel were fixed with methanol and stained with 0.2% crystal violet, and then quantified using four random fields under a microscope.

### 4.5. Co-Immunoprecipitation (CoIP) Assay

Cells were collected and lysed with IP lysates on ice for 40 min, and then centrifuged at 13,000 rpm for 4 min at 40 °C. The supernatant was collected and the protein concentration was measured using the BCA method. Immunoglobulin G (IgG, 2 μg) was added to 1 mg of protein, mixed with 20 μL of protein A/G Sepharose beads for 1 h in a homomixer, and then centrifuged at 2500 rpm for 5 min. The supernatant was collected, 1 mg of protein was added to 2 μg of the target protein antibody, and the mixture was mixed overnight. Protein A/G Sepharose (20 μL) were added to the protein and mixed for 4 h in a homomixer. Beads were washed with phosphate-buffered saline (PBS) three times, the supernatants were collected, and Western blot analysis was conducted.

### 4.6. In Vivo Tumor Metastatic Assay 

Female nude mice of 4–6 weeks old were purchased, 10 in each group. The cells were collected, washed 2–3 times with PBS, and then resuspended in PBS. A total of 1 × 10^6^ ESCC cells were injected intravenously into the animals via the tail vein. The weight of nude mice was measured weekly. Metastasis was monitored weekly using bioluminescent imaging (Xenogen IVIS Lumima II, PerkinElmer, Waltham, MA, USA). Their care was in accordance with institution guidelines. This study was approved by the Institutional Review Board of the Jinan University (ethical code: 20180705-19, 5 July 2018) and performed in accordance with the Declaration of Basel (American Journal of Health-System Pharmacy. 2009, 4; 31 August 2008).

### 4.7. Statistical Analysis

All in vitro experiments and assays were repeated at least three times. Statistically significant differences were calculated using a Student’s *t*-test method that was performed using GraphPad Prism 6.0 (La Jolla, CA, USA). Statistical significance was defined as *p* < 0.05 (*, *p* < 0.05; **, *p* < 0.01; ***, *p* < 0.001).

## 5. Conclusions

This report reveals that RNF128 plays a functional role in driving the invasion and metastasis of ESCC through the EGFR/MAPK/MMP-2 pathway. RNF128 promotes the activation of the EGFR/MAPK/MMP-2 pathway by interacting with p53 and p53 interacting with EGFR. RNF128 is a potential candidate to be further explored as an anti-metastasis target.

## Supplementary Materials

The following are available online at https://www.mdpi.com/2072-6694/11/6/840/s1, Figure S1: The expression of RNF128 and its function in clone formation, Figure S2: Transcriptome sequencing and bioinformatics analyses identified RNF128-regulated genes and pathways, Figure S3: The whole blot showing all the bands with all molecular weight markers on the Western blotting, Figure S4: The whole blot showing all the bands with all molecular weight markers on the Western blotting.
