# Supplementary material for: RNF128 Promotes Invasion and Metastasis Via the EGFR/MAPK/MMP-2 Pathway in Esophageal Squamous Cell Carcinoma
Source: Cancers (Basel). 2019 Jun 18;11(6):840. doi: 10.3390/cancers11060840 (PMC6627419; doi:10.3390/cancers11060840)
Supplement: Supplementary file 1 [file cancers-11-00840-s001.pdf]

Supplementary Materials: RNF128 Promotes Invasion and Metastasis Via the EGFR/MAPK/MMP-2 Pathway in Esophageal Squamous Cell Carcinoma

Jing Gao, Yang Wang, Jie Yang, Weixia Zhang, Kun Meng, Yue Sun, Yangjia Li and Qing-Yu He

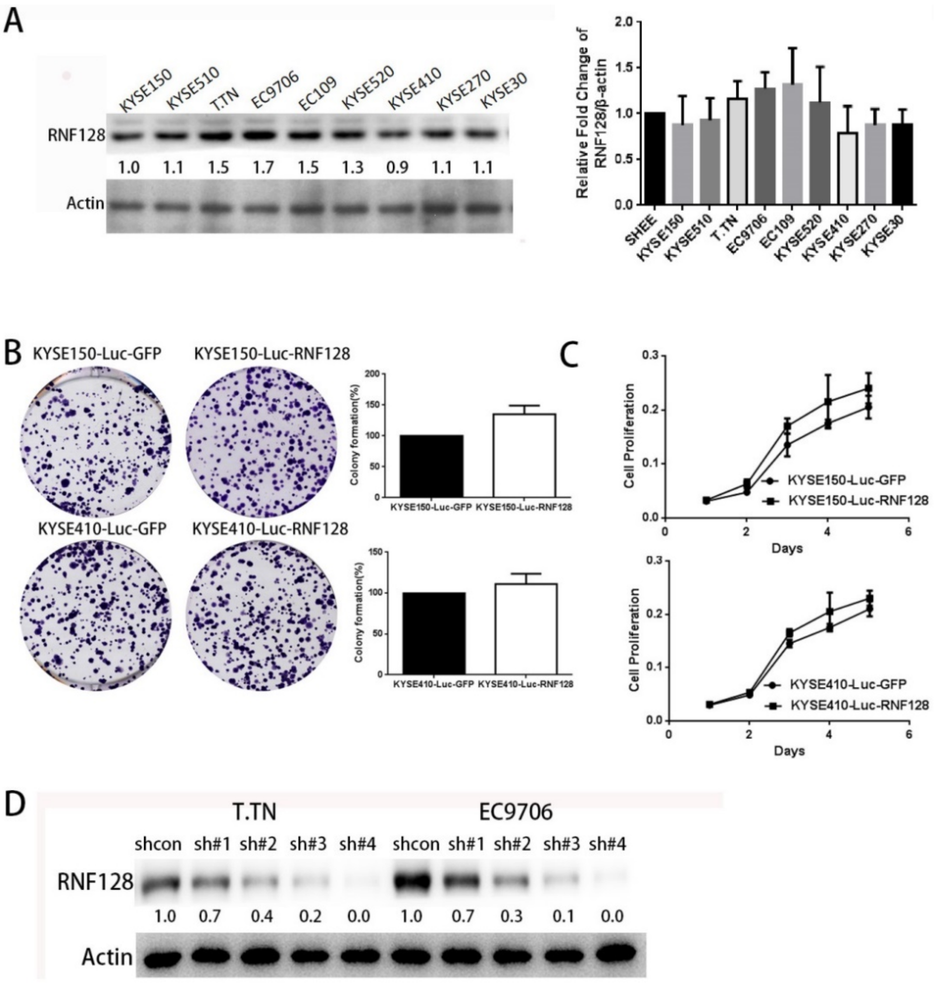

Figure S1. The expression of RNF128 and its function in clone formation. (**A**) Western blotting was performed to detect the expression of RNF128 in 9 ESCC cell lines including EC109, KYSE270 and T.Tn with low-metastatic grade, and KYSE30, KYSE520 and EC9706 with higher-metastatic grade. (**B**) Cloning assay and quantification with RNF128 overexpressing and control cells. (**C**) Five consecutive days of WST-1 detection in RNF128 overexpressing and control cells. (**D**) Validation of RNF128 knockdown at protein levels by Western blotting.

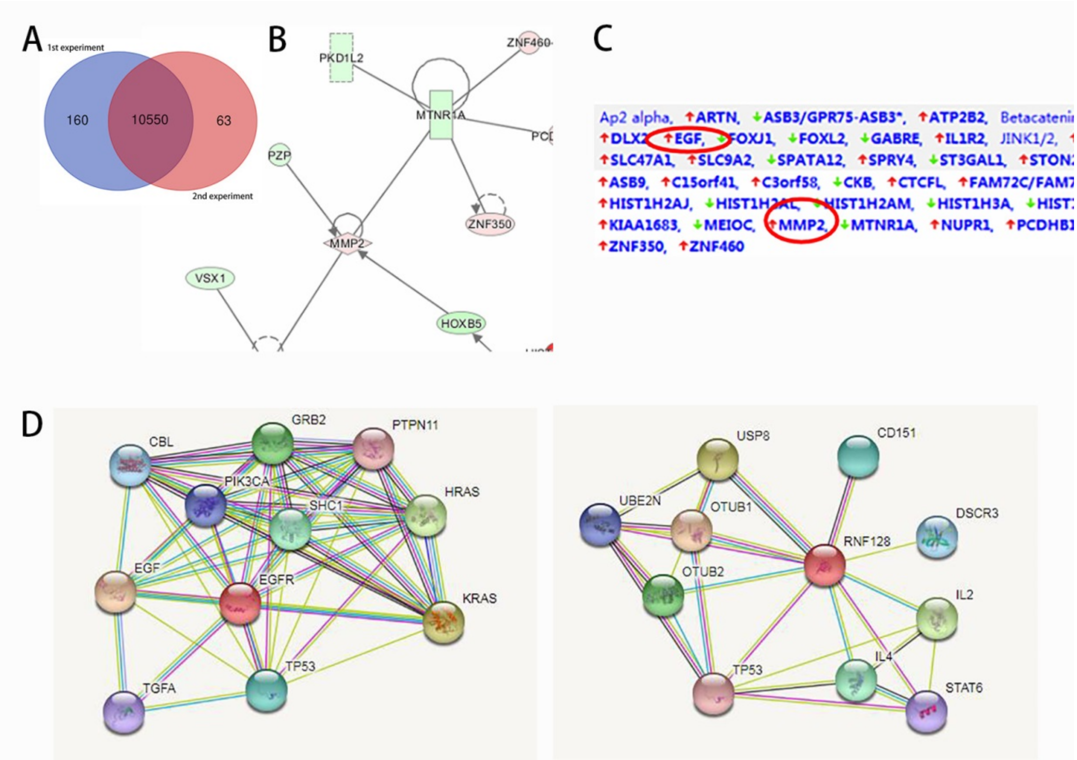

Figure S2. Transcriptome sequencing and bioinformatics analyses identified RNF128-regulated genes and pathways. (**A**) Venn diagram showing the number of overlapped genes in two experiments. (**B**,**C**) The differentially expressed genes by IPA analysis in RNF128-overexpressing KYSE150 and KYSE150-Luc cells. (**D**) p53 was found to interact with RNF128 and EGFR by STRING analysis.

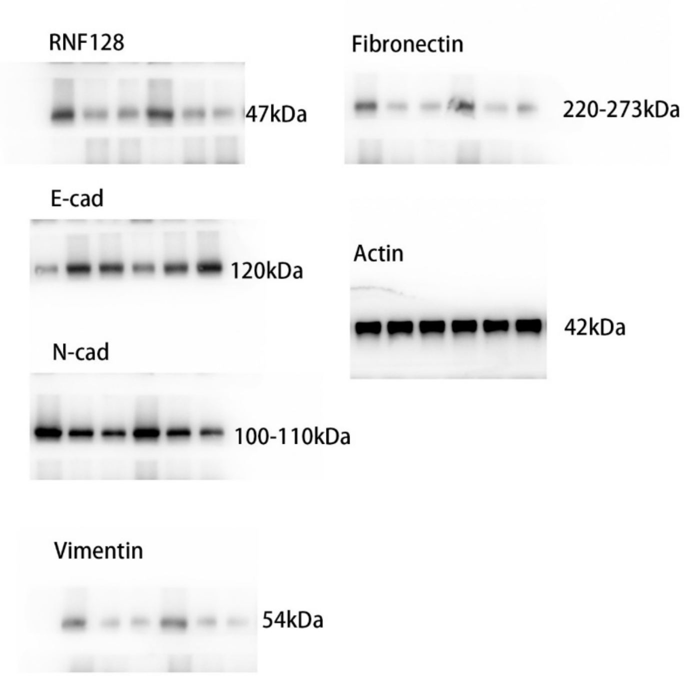

Figure S3. The whole blot showing all the bands with all molecular weight markers on the Western blotting.

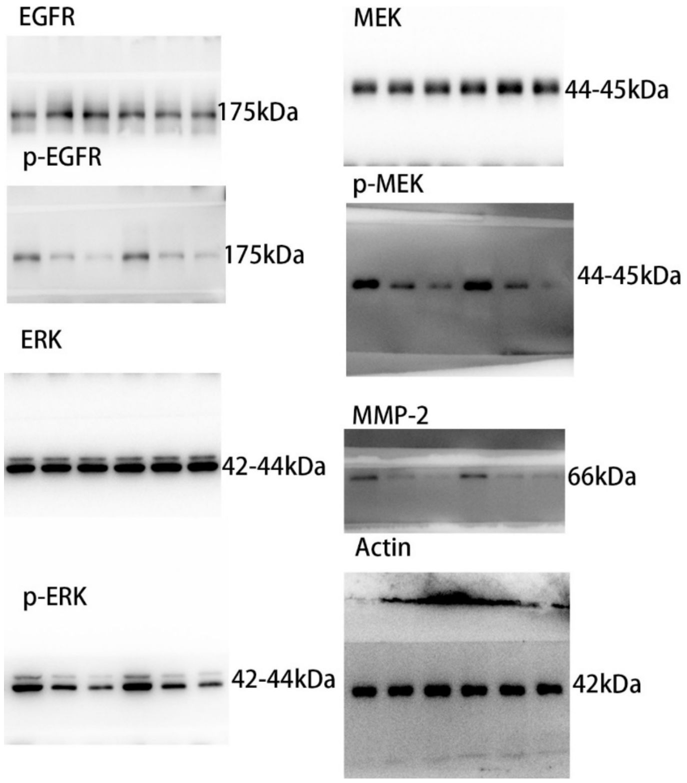

Figure S4. The whole blot showing all the bands with all molecular weight markers on the Western blotting.

© 2019 by the authors. Licensee MDPI, Basel, Switzerland. This article is an open access article distributed under the terms and conditions of the Creative Commons Attribution (CC BY) license (http://creativecommons.org/licenses/by/4.0/).
